# Investigating health-related quality of life in rare diseases: a case study in utility value determination for patients with CLN2 disease (neuronal ceroid lipofuscinosis type 2)

**DOI:** 10.1186/s13023-021-01829-x

**Published:** 2021-05-12

**Authors:** Paul Gissen, Nicola Specchio, Andrew Olaye, Mohit Jain, Thomas Butt, Wrik Ghosh, Benjamin Ruban-Fell, Annabel Griffiths, Charlotte Camp, Zlatko Sisic, Christoph Schwering, Eva Wibbeler, Marina Trivisano, Laura Lee, Miriam Nickel, Amanda Mortensen, Angela Schulz

**Affiliations:** 1 NIHR Biomedical Research Centre, UCL Great Ormond Street Institute of Child Health, 30 Guilford Street, London, WC1N 1EH UK; 2 Department of Neuroscience, Bambino Gesù Children’s Hospital, IRCCS, Rome, Italy; 3 BioMarin Europe Ltd, London, UK; 4 Costello Medical, Singapore, Singapore; 5 Costello Medical, Cambridge, UK; 6 Children’s Hospital, University Medical Center Hamburg-Eppendorf, Hamburg, Germany; 7 Department of Metabolic Medicine, Great Ormond Street Hospital, London, UK; 8 Department of Pediatrics, University Medical Center Hamburg-Eppendorf, Hamburg, Germany; 9 Batten Disease Family Association, London, UK

**Keywords:** CLN2, Utility values, Vignettes, Cerliponase alfa

## Abstract

**Background:**

Utility studies enable preference-based quantification of a disease’s impact on patients’ health-related quality of life (HRQoL). It is often difficult to obtain utility values for rare, neurodegenerative conditions due to cognitive burden of direct elicitation methods, and the limited size of patient/caregiver populations. CLN2 disease (neuronal ceroid lipofuscinosis type 2) is an ultra-rare, progressive condition, for which there are no published utility data fully capturing all disease stages. This case study demonstrates how utility values can be estimated for ultra-rare paediatric diseases by asking clinicians to complete EQ-5D-5L questionnaires based on vignettes describing the stages of CLN2 disease.

**Methods:**

An indirect elicitation method using proxy-reporting by clinical experts was adopted. Eighteen vignettes were developed, describing nine progressive disease stages as defined by motor and language domain scores of the CLN2 Clinical Rating Scale, in individuals treated with cerliponase alfa or standard care. Eight clinical experts with experience of treating CLN2 disease with cerliponase alfa and current standard care completed the proxy version 2 EQ-5D-5L online after reading these vignettes. Resulting scores were converted to EQ-5D-5L utility values for each disease stage, using UK, German and Spanish value sets.

**Results:**

Utility values, which are typically anchored by 0 (equivalent to death) and 1 (full health), decreased with CLN2 disease progression (results spanned the maximum range of the utility scale). Assigned utility values were consistently higher for patients receiving cerliponase alfa than standard care; differences were statistically significant for the 6 most severe disease stages (*p* < 0.05). Analysis of the individual dimensions of the EQ-5D-5L showed that greatest differences between patients treated with cerliponase alfa and standard care occurred in the pain dimension (differences in mean scores ranged between no difference and 1.8), with notable differences also observed in the anxiety/depression dimension (differences in mean scores ranged between 0.1 and 1.0).

**Conclusions:**

This study demonstrates a feasible methodology for eliciting utility values in CLN2 disease, indicating HRQoL declines with disease progression. Vignettes describing patients receiving cerliponase alfa were consistently assigned higher utility values for the same disease state, suggesting this treatment improves HRQoL compared with standard care.

*Trial registration* NCT01907087, NCT02485899.

**Supplementary Information:**

The online version contains supplementary material available at 10.1186/s13023-021-01829-x.

## Introduction

Utility values are used to quantify the impact of a disease on patients’ health-related quality of life (HRQoL) and are an important component of evidence generation in disease-related research. Utilities express individuals’ preferences for different health states, and enable the impact of different diseases and interventions on HRQoL to be compared using a common metric [1]. They can therefore be used to generate quality-adjusted life years (QALYs), a summary measure of health outcomes incorporating the impact of a treatment on both the quantity and quality of life [2]. QALYs are calculated by estimating the years of life remaining for a patient and weighting each year with a QoL score [3, 4]. Utility values are measured on an interval scale, with zero equivalent to death, and one equivalent to perfect health. A utility score of less than zero represents a health state considered to be worse than death.

Obtaining utility values in rare diseases is often challenging due to a number of issues. Small patient population sizes may result in insufficient data to draw meaningful or robust conclusions, particularly in studies aimed at eliciting disease stage-specific values, which require further subdivisions of the patient population. In rare, neurodegenerative diseases, neurocognitive symptoms may make it challenging for patients to participate in utility collection studies. Furthermore, the majority of rare diseases are paediatric, and eliciting utility values from children is itself associated with a number of challenges and ethical considerations [5, 6]. Whilst proxy-reported health measures are often completed by parents, their experience is likely to be limited to that of their own child. Consequently, it may be more appropriate to use clinical experts who have a wider range of experience with such patients. Despite this, the rarity of these diseases may result in clinicians having a lack of knowledge or experience in treating these conditions, leading to a small number of clinicians with sufficient knowledge of the disease to advise or participate in studies.

The use of vignettes to derive utility values is a methodological approach in which brief descriptions of health states for a disease are developed, which are then assessed using a preference-based measure, such as the EQ-5D questionnaire [7]. This technique is often used in the absence of data derived from validated HRQoL methods, which can help to overcome the challenges associated with small just population sizes [7]. The use of vignettes offers several advantages in the development of economic models; this technique can provide a detailed hypothetical situation with no requirement for patient level data [7]. A key methodological strength of vignettes in the context of this study is that they also allow indirect elicitation of patients’ preferences for different disease states where otherwise HRQoL data collection in certain health states would be considered unethical or impractical (e.g. due to patients’ level of cognition). Additionally, vignettes can be designed to incorporate concerns and side-effects of treatment that are of importance to patients [7].

CLN2 disease (neuronal ceroid lipofuscinosis type 2) is an ultra-rare, autosomal recessive, neurodegenerative disorder with an estimated prevalence of 0.75 per million and incidence of 0.5 per 100,000 live births [8, 9]. This varies by country, with incidences ranging from 0.15 per 100,000 live births in Portugal, to 0.78 per 100,000 live births in the United Kingdom (UK), for example [8]. In the UK, there are approximately 30–50 children living with CLN2 disease, with 3–6 diagnosed each year [10].

CLN2 disease is caused by mutations in the *CLN2/TPP1* gene resulting in tripeptidyl peptidase-1 (TPP1) deficiency [11]. TPP1 deficiency results in lysosomal accumulation of ceroid lipofuscin, and is associated with progressive and selective neuronal and retinal cell loss [11, 12]. CLN2 disease typically has late-infantile onset and follows a predictable clinical course, characterised by epilepsy and declining psychomotor function [13]. Progression of CLN2 disease is rapid, eventually resulting in loss of language, independent mobility, and vision [14]. Patients with CLN2 disease experience early death, with a median life expectancy reported as 10.1 years [14].

The current standard care for CLN2 disease is symptomatic and palliative, focusing on management of generalised tonic–clonic seizures (GTCSs; a seizure that produces bilateral convulsive tonic and clonic muscle contractions), and loss of motor control and feeding [15]. Cerliponase alfa, a recombinant form of human TPP1, has been approved by a number of regulatory bodies, including the European Medicines Agency (EMA) and the Food and Drug Administration (FDA), for the treatment of CLN2 disease [16]. Treatment with cerliponase alfa has been shown to slow progression of CLN2 disease [16]. Two clinical trials: an open label, single group study (Study 190–201; NCT01907087) and an extension study (Study 190–202; NCT02485899), have demonstrated a clinically significant difference between cerliponase alfa treatment and historical controls; treatment with cerliponase alfa was shown to result in a slower rate of decline of motor and language function in CLN2 disease patients. The extension study (Study 190–202) assessing the long-term use of cerliponase alfa is ongoing [17]. Whilst HRQoL was included as an exploratory efficacy outcome of these studies, further data are required to understand the impact of the different stages of CLN2 disease on HRQoL.

Currently, no utility values have been collected that fully represent the different stages of CLN2 disease. The aim of this study was to obtain utility values for the different stages of CLN2 disease, for patients treated with cerliponase alfa or with current standard care (symptomatic treatment as described above), to provide insight into the impact of cerliponase alfa on HRQoL in patients with CLN2 disease and to provide a case study for utility value generation in a rare condition.

## Methods

### Vignette development

Nine progressive disease stages were described in the English language and were informed by discussions held prior to vignette development with clinical experts who had experience of treating CLN2 disease with standard care and cerliponase alfa. Motor and language domain scores, as measured by the CLN2 Clinical Rating Scale (a validated, disease-specific measure adapted from the common subscales of the pre-existing Hamburg and Weill Cornell scales, to be used as an assessment tool for multicentre efficacy studies supporting the development of cerliponase alfa [18]), were used to define the various disease stages [19]. Descriptions of motor and language within the vignettes were aligned with the definitions used in the CLN2 Clinical Rating Scale to guide clinicians’ interpretations of the vignettes. The progressive disease stages represented each of the possible scores of the motor and language domains of the CLN2 Clinical Rating Scale (0–6) plus two more severe stages of disease: the first where vision has been completely lost, the second where patients are also receiving palliative care. Details of other progressive symptoms which may influence the patient experience but were not covered as part of the motor and language domains, were included to further capture the clinical reality of disease progression. These included vision, chronic GTCSs, disease-related distress, dystonia (abnormalities of muscle tone resulting in muscular spasms), myoclonus (sudden, uncontrolled muscle twitching with multiple contractions that can occur in different parts of the body), and use of a feeding tube. Descriptions of the nature of these progressive symptoms within the vignettes (excluding motor and language) differed between those describing treated and untreated patients, based on the guidance of clinical experts. Additional information describing the nature of cerliponase alfa treatment was also added to vignettes describing treated patients. It should be noted that symptoms and progression of CLN2 disease follow a typically uniform path. Given that variability between patients in the same disease state is therefore likely to be minimal, the use of vignettes in this study was deemed to be appropriate, on the assumption that these vignettes encapsulate the clinical outcomes of ‘typical’ patients with CLN2 disease [14].

On the CLN2 Clinical Rating Scale, a maximum score of six can be obtained by achieving a score of three in each of the two domains (motor and language); an overall score of zero is obtained with a score of zero in both domains (Additional file 1: Table S1). As the disease progresses, patients lose function and progress to lower scores. Each vignette described the most common combination of motor and language domain scores (as determined by clinical experts’ opinion [PG]) that gave the relevant CLN2 Clinical Rating Scale score for that disease stage. For example, a CLN2 Clinical Rating Scale score of 3 is most commonly made up of a motor score of 1 and a language score of 2 (Table 1). A summary of the disease stages and terminology used in this manuscript is given in Table 1. In total, 18 vignettes were developed corresponding to each of the disease stages and describing patients treated with either cerliponase alfa or standard care in each respective stage.

**Table 1:** Summary of disease stages

Disease stage	CLN2 clinical rating scale score (motor score + language score)	Additional characteristics
6	6 (3 + 3)	None
5	5 (2 + 3)	None
4	4 (2 + 2)	None
3	3 (1 + 2)	None
2	2 (1 + 1)	None
1	1 (1 + 0)	None
0	0	None
0 + VL	0	With vision loss
0 + VL + PC	0	With vision loss and requiring palliative care

Disease progression increases with decreasing disease stage

CLN2, neuronal ceroid lipofuscinosis type 2; PC, palliative care; VL, vision loss

Vignette validation was performed by a single clinical expert (PG), with significant experience of CLN2 disease and cerliponase alfa. In addition, vignettes were reviewed by an external utility collection expert. The vignettes can be found in full in Supplementary Information 1.

### Study participation

Eight clinical experts, all of whom had experience with both CLN2 patients and cerliponase alfa treatment, participated in this study. The clinical experts were selected from three European treatment centres to increase geographic representation and prevent bias from being introduced by one centre: Great Ormond Street Hospital (UK; PG, LL), Bambino Gesù Children’s Hospital (Italy; NS, MT), and University Medical Centre Hamburg-Eppendorf (Germany; CS, EW, MN, AS). At the time of the study, these were the only centres in Europe where clinicians had experience of treating CLN2 disease with cerliponase alfa.

### Questionnaire-based generation of utility values

The EuroQol EQ-5D-5L is a preference-based measure used to determine utility values, consisting of five dimensions (mobility, self-care, usual activities, pain/discomfort and anxiety/depression) and five levels (no problems, slight problems, moderate problems, severe problems and extreme problems) [20]. An online version of the proxy version 2 EQ-5D-5L questionnaire was used and approved for use by EuroQol (Additional file 1: Figure S1). This version of the questionnaire instructs participants to: ‘rate how he/she (the proxy) thinks the patient would rate his/her own health-related quality of life, if the patient were able to communicate it’ [20].

Following distribution of vignettes via email, study participants independently completed the questionnaire for each vignette as proxies for CLN2 patients experiencing the disease stages described in each of the vignettes. Participants were requested to complete the questionnaire within two weeks of receiving the vignettes. Participants were presented with the vignettes for patients treated with standard care first, in increasing order of disease progression, followed by the vignettes for patients treated with cerliponase alfa in the same order. The clinician who validated the vignettes was also asked to complete the questionnaire as a proxy for CLN2 patients. In order to control for any bias introduced by this decision, sensitivity analyses were conducted, in which results from the clinical expert involved in vignette validation were removed.

Using a value set for the UK general public [21], proxy EQ-5D-5L responses were converted to utility values. From these EQ-5D-5L utility values, EQ-5D-3L utility values were derived using the crosswalk methodology developed by Van Hout et al., in line with preferences stated by the National Institute for Health and Care Excellence (NICE) [22, 23].

As the clinical experts were based in the UK, Germany and Italy, German and Spanish (in the absence of a suitable Italian alternative) EQ-5D-5L value sets were used to convert EQ-5D-5L responses into utility values for the German and Italian populations, respectively [24, 25]. Whilst an Italian EQ-5D-3L value set exists, no Italian EQ-5D-5L value set or crosswalk methodologies are available and subsequently, a Spanish EQ-5D-5L set was used to convert EQ-5D-5L responses into utility values for the Italian population. The validity of this approach has been demonstrated previously [26].

Paired t-tests were conducted to assess the statistical significance of differences between mean utility values of standard care and cerliponase alfa for different health states. Paired t-tests were chosen because (a) differences between utility values for patients treated with cerliponase alfa and standard care were unlikely to be independent from one another and (b) a parametric test was deemed to have sufficient power to detect significance (in light of the small size of the dataset). In order to assess the level of agreement between participants answering the questionnaire (known as inter-rater agreement), average intraclass correlation (ICC) values were obtained by applying a two-way, mixed consistency approach to each vignette [27]. Average ICC values between 0.75 and 1.00 are commonly interpreted to demonstrate excellent inter-rater agreement [27].

## Results

### EQ-5D-5L derived UK utility values

**Fig. 1:**
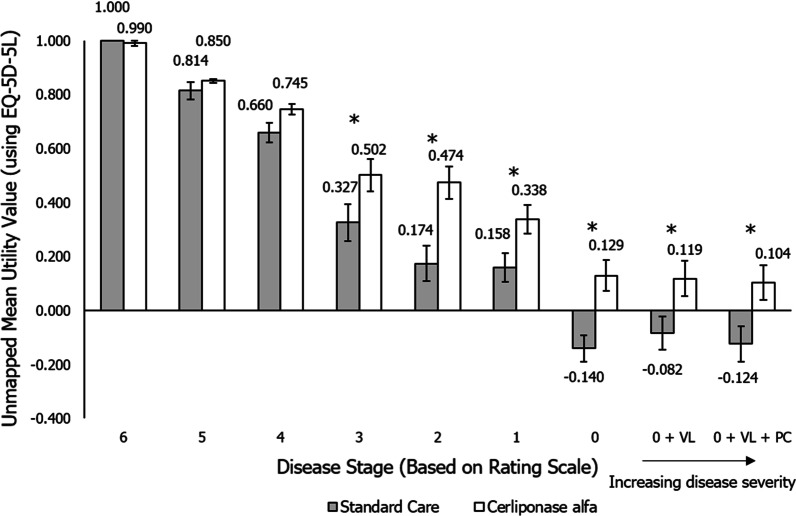
Mean utility values across disease stages using the unmapped EQ-5D-5L value set. Mean values ± 1 standard error are shown on the chart. Utility values are given on a scale where 1 is equivalent to perfect health, and 0 equivalent to death. UK value set. Asterisks (*) indicate a statistically significant difference between treatment with standard care and cerliponase alfa using a paired t-test, *p* < 0.05. VL, vision loss; PC, palliative care

Utility values, based on the EQ-5D-5L responses from the eight participants for vignettes describing all nine disease stages for both patients treated with standard care and those treated with cerliponase alfa, were obtained using the UK value set. In patients with a CLN2 Clinical Rating Scale score of 6 (the least impaired disease stage), utility values were equivalent to perfect health and similar for vignettes describing patients receiving cerliponase alfa treatment compared to treatment with standard care, with mean (standard error) values of 0.990 (0.010) and 1.000 (0.000), respectively (Tables 2, 3, Fig. 1). Utility values then decreased as the vignettes described increasingly more progressed stages of disease.

**Table 2:** Utility values of patients treated with cerliponase alfa using the UK EQ-5D-5L value set

Disease stage	Cerliponase alfa				
	Mean value	Standard error	Median value	Minimum value	Maximum value
6	0.990	0.010	1.000	0.924	1.000
5	0.850	0.008	0.846	0.825	0.901
4	0.745	0.019	0.761	0.642	0.801
3	0.502	0.061	0.539	0.302	0.666
2	0.474	0.060	0.436	0.267	0.666
1	0.338	0.053	0.317	0.167	0.652
0	0.129	0.057	0.179	− 0.213	0.282
0 + VL	0.119	0.065	0.186	− 0.281	0.282
0 + VL + PC	0.104	0.065	0.174	− 0.281	0.268

Disease progression increases with decreasing disease stage. Utility values are given on a scale where 1 is equivalent to perfect health, and 0 equivalent to death. UK value set

PC, palliative care; VL, vision loss

**Table 3:** Utility values of patients treated with standard care using the UK EQ-5D-5L value set

Disease stage	Standard care				
	Mean value	Standard error	Median value	Minimum value	Maximum value
6	1.000	0.000	1.000	1.000	1.000
5	0.814	0.032	0.846	0.608	0.901
4	0.660	0.036	0.665	0.447	0.801
3	0.327	0.069	0.367	− 0.102	0.522
2	0.174	0.065	0.261	− 0.137	0.329
1	0.158	0.053	0.228	− 0.137	0.329
0	− 0.140	0.049	− 0.206	− 0.276	0.073
0 + VL	− 0.082	0.061	− 0.120	− 0.276	0.206
0 + VL + PC	− 0.124	0.066	− 0.213	− 0.281	0.191

Disease progression increases with decreasing disease stage. Utility values are given on a scale where 1 is equivalent to perfect health, and 0 equivalent to death. UK value set

PC, palliative care; VL, vision loss

In the final disease stages, where the CLN2 Clinical Rating Scale score reaches zero, the mean utility values fell below zero for vignettes describing patients treated with standard care. For patients assigned a CLN2 Clinical Rating Scale score of 0, 0 + vision loss (VL), and 0 + VL + palliative care (PC), the mean (standard error) utility values for vignettes describing patients treated with standard care were − 0.140 (0.049), − 0.082 (0.061) and − 0.124 (0.066), respectively. Negative utility values were not observed in the equivalent disease stages for patients treated with cerliponase alfa; the mean (standard error) utility values for these disease stages were 0.129 (0.057), 0.119 (0.065), and 0.104 (0.065), respectively.

Utility values for vignettes describing patients treated with cerliponase alfa were higher than for patients treated with standard care in the more progressed stages of the disease (patients with a CLN2 Clinical Rating Scale score of 5 and below; Fig. 1), with the difference between the equivalent mean scores at each disease stage ranging between 0.01–0.30. Statistically significant differences between utility values for vignettes describing patients treated with cerliponase alfa and with standard care were observed for disease stages 0 + VL + PC, 0 + VL and stages 0–3, with *p* values ranging from 0.002 to 0.045 (Fig. 1).

ICC values demonstrated excellent inter-reader agreement between vignettes (Table 4).

**Table 4:** ICC results for UK, German and Spanish datasets

Dataset	ICC	Interpretation
UK	0.983	Excellent
German	0.981	Excellent
Spanish	0.985	Excellent

ICC interpretations: below 0.40: poor; 0.40–0.59: fair; 0.60–0.74: good; 0.75–1.00: excellent. A Spanish value set was used as a substitute for the Italian population in the absence of an available Italian value set

ICC, intraclass correlation

### EQ-5D-5L dimension analyses

**Fig. 2:**
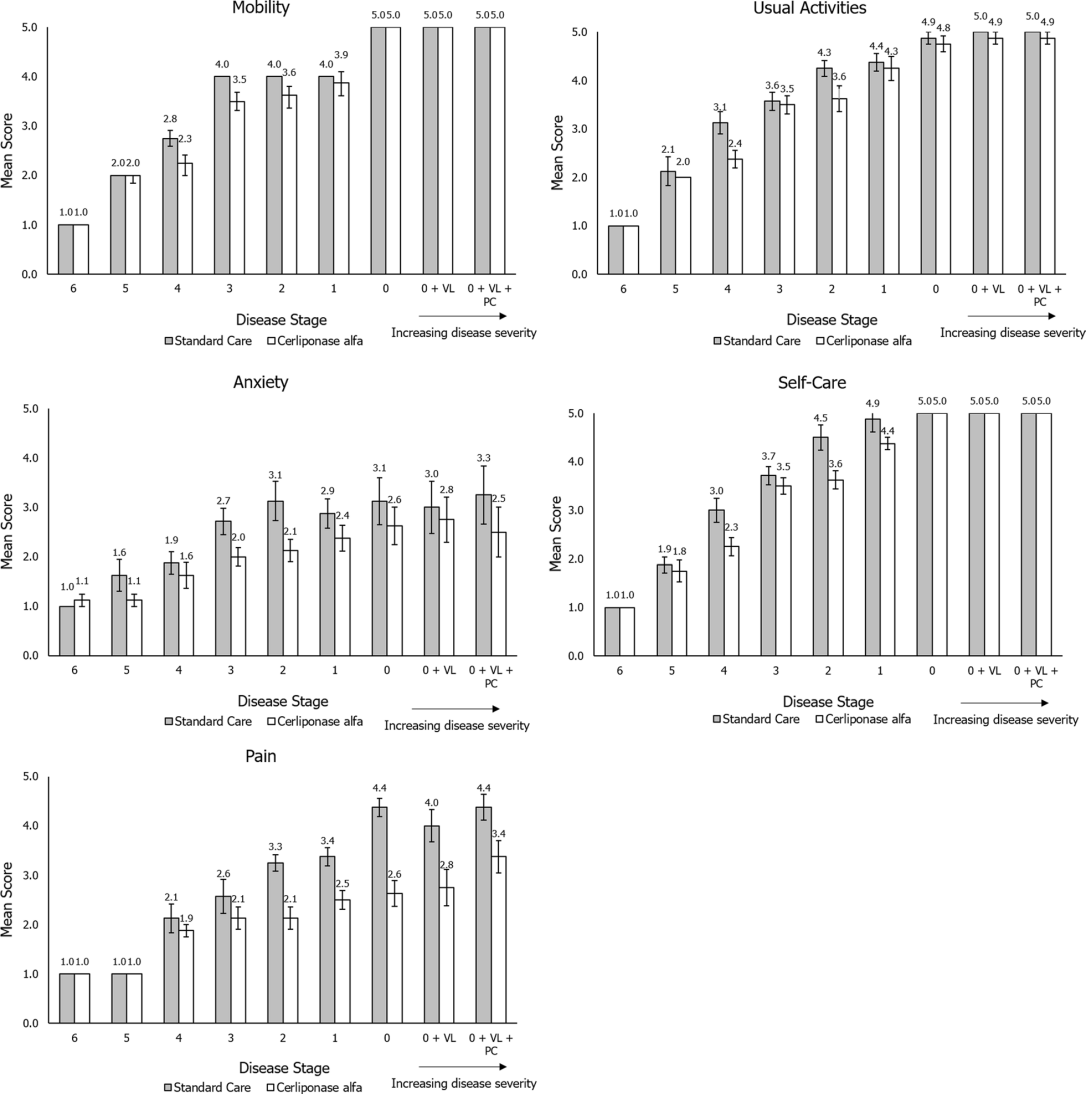
Mean score across disease stages in individual dimensions of the EQ-5D-5L. 1 = no problems, 2 = slight problems, 3 = moderate problems, 4 = severe problems, 5 = extreme problems. Mean values ± 1 standard error are shown on the chart. UK value set. VL, vision loss; PC, palliative care

Additional analyses were carried out on the individual dimensions of the proxy EQ-5D-5L to determine which dimensions were primarily responsible for the differences in utility values observed between patients treated with cerliponase alfa and standard care (Fig. 2). Notable differences between the mean scores of patients treated with cerliponase alfa and with standard care were present in the pain/discomfort dimension, with patients treated with cerliponase alfa having consistently lower mean scores across the nine disease stages. Similarly, notable differences were observed in the anxiety/depression dimension. The mean scores for the other dimensions of the proxy EQ-5D (mobility, self-care and usual activities) were marginally lower for vignettes describing patients treated with cerliponase alfa compared with standard care, with largest differences observed for diseases stages 1–4.

### UK utility values mapped to the EQ-5D-3L

**Fig. 3:**
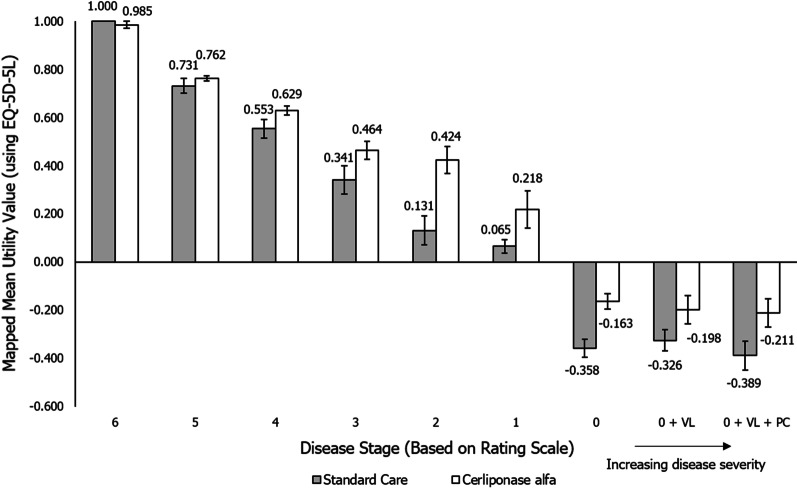
Mean utility values of patients treated with cerliponase alfa and standard care (mapped to EQ-5D-3L). Summary values were produced from the returned EQ-5D-5L questionnaires and results were mapped to equivalent EQ-5D-3L utility values using the Van Hout algorithm [23]. Mean values ± 1 standard error are shown on the chart. Utility values are given on a scale where 1 is equivalent to perfect health, and 0 equivalent to death. UK value set. VL, vision loss; PC, palliative care

Results were mostly unaffected by mapping values to the EQ-5D-3L (Table 5, 6, Fig. 3) using the crosswalk methodology, with the difference between the mapped and unmapped scores at each disease stage ranging between 0.000 and 0.317 [22]. However, one key difference was that utility values for vignettes describing patients treated with cerliponase alfa fell below zero in the later disease stages, in contrast to the corresponding EQ-5D-5L values.

**Table 5:** Utility values of patients treated with cerliponase alfa with mapping to the EQ-5D-3L

Disease stage	Cerliponase alfa					
	Mean value	Standard error	Median value	Minimum value	Maximum value	Difference between EQ-5D-5L and mapped EQ-5D-3L utility values
6	0.985	0.015	1.000	0.879	1.000	0.005
5	0.762	0.011	0.747	0.747	0.836	0.088
4	0.629	0.019	0.636	0.541	0.710	0.116
3	0.464	0.036	0.510	0.325	0.555	0.038
2	0.424	0.056	0.468	0.101	0.555	0.050
1	0.218	0.078	0.267	− 0.166	0.531	0.120
0	− 0.163	0.033	− 0.159	− 0.352	− 0.071	0.292
0 + VL	− 0.198	0.060	− 0.147	− 0.594	− 0.071	0.317
0 + VL + PC	− 0.211	0.058	− 0.154	− 0.594	− 0.095	0.315

Disease progression increases with decreasing disease stage. Utility values are given on a scale where 1 is equivalent to perfect health, and 0 equivalent to death. Summary values were produced from the returned EQ-5D-5L questionnaires and results were mapped to equivalent EQ-5D-3L utility values using the Van Hout algorithm [23]. UK value set

PC, palliative care; VL, vision loss

**Table 6:** Utility values of patients treated with standard care with mapping to the EQ-5D-3L

Disease stage	Standard care					
	Mean value	Standard error	Median value	Minimum value	Maximum value	Difference between EQ-5D-5L and mapped EQ-5D-3L utility values
6	1.000	0.000	1.000	1.000	1.000	0.000
5	0.731	0.031	0.747	0.544	0.836	0.083
4	0.553	0.038	0.549	0.353	0.710	0.107
3	0.341	0.058	0.371	0.036	0.518	0.014
2	0.131	0.059	0.116	− 0.087	0.310	0.043
1	0.065	0.028	0.081	− 0.086	0.155	0.093
0	− 0.358	0.038	− 0.352	− 0.510	− 0.200	0.218
0 + VL	− 0.326	0.044	− 0.352	− 0.510	− 0.127	0.244
0 + VL + PC	− 0.389	0.059	− 0.355	− 0.594	− 0.151	0.265

Disease progression increases with decreasing disease stage. Utility values are given on a scale where 1 is equivalent to perfect health, and 0 equivalent to death. Summary values were produced from the returned EQ-5D-5L questionnaires and results were mapped to equivalent EQ-5D-3L utility values using the Van Hout algorithm [23]. UK value set

PC, palliative care; VL, vision loss

### Exclusion of the clinical expert involved in vignette validation

Furthermore, following an adjustment of the EQ-5D-5L and EQ-5D-3L analyses to exclude the responses of the clinical expert involved in vignette validation, results were relatively unchanged (Additional file 1: Tables S2–S5, Additional file 1: Figures S2 and S3).

### EQ-5D-5L derived German and Spanish utility values

Use of German and Spanish EQ-5D-5L value sets demonstrated minimal differences in utility values between the three geographic regions (Additional file 1: Tables S6–S9, Additional file 1: Figures S4 and S5) [24, 26].

## Discussion

The purpose of this study was to obtain utility values for the different stages of CLN2 disease, for patients treated with cerliponase alfa or standard care, providing insight into the impact of cerliponase alfa treatment on patients' HRQoL. The results of this study, which considered patients with a classical (non-atypical) disease phenotype, indicate that utility values, and thus HRQoL, decrease with progression to more severe stages of the disease. The techniques described in this study have been previously used in areas where utility values were not available in the literature [28–32], and were considered to be an appropriate methodology for obtaining utility values in a disease of this nature.

Previous literature has suggested that, where possible, direct elicitation from the patient should be the primary source of HRQoL information when estimating utility values [33, 34]. However, a valid and reliable preference-based measure of utility for health states that can be used in children of all ages is not available, and it is acknowledged that the abstract elements contained in the EQ-5D pose challenges of understanding for young children [35]. Subsequently, it is considered that for children aged < 6 years, involvement of a proxy such as a clinician or carer is the most feasible method for capturing substantive HRQoL information, and therefore use of the proxy version of the EQ-5D questionnaire was deemed appropriate for the purposes of this study.

However, the usefulness of adult proxies in the determination of paediatric utility values must be considered in relation to the complexities of judging HRQoL from a child’s perspective. HRQoL has been described as a multidimensional construct that includes physical, psychological and social domains [36]; given that these aspects of a child’s life differ somewhat from those of an adult, it is possible that clinicians completing the questionnaires in this study may have influenced results by projecting their own perceptions of the patient’s HRQoL or that of the patient’s parents [37]. When completing objective and observable metrics (an evaluation of a patient’s physical functioning, for example), assessments by proxy raters show a strong degree of uniformity relative to those same metrics garnered from direct patient elicitation; in contrast, for psychosocial and other assessments with a perceived level of subjectivity (for example, mental health or social functioning), greater divergence in responses is frequently observed [38, 39]. Parents and caregivers are well-placed to assess their child’s day-to-day behaviour and social interactions outside of the clinical setting, and any small changes in these aspects through the disease stages may be detected more reliably. On a case-by-case basis, therefore, caregivers may be better able to interpret the psychosocial aspects of the vignettes and their child’s scoring in the EQ-5D questionnaire.

Nevertheless, additional challenges arise when considering the use of parent proxy reports of HRQoL in CLN2 disease. The probability of identifying a sufficient sample of parents across the disease stages, for both treated and untreated children, was limited for this study due to the rarity of the disease. Parents of a long-deceased child may be affected by recall bias, particularly when asked to evaluate their child’s HRQoL at multiple disease stages. In the case of parents with a living child with CLN2 disease, perceptions of their child’s HRQoL may be unduly influenced by their own coping strategies, such as the extent of their acceptance or denial of their child’s illness (a feature observed in studies of parent proxy reports of HRQoL in children with Duchenne Muscular Dystrophy) [39, 40]. The overall breadth of CLN2 experience among clinical experts in managing both treated and untreated children across all disease stages deemed their use as proxies to be the most appropriate way of obtaining EQ-5D-based utility values in this population.

Previously, it has been acknowledged that clinicians and caregivers tend, in general, to underestimate patients’ quality of life [41]. Nonetheless, a ‘perfect health’ mean utility value of 1.0 was observed for the disease stage represented by a CLN2 Clinical Rating Scale score of 6 in patients treated with standard care, in keeping with the vignette description that suggests a patient without problems in any of the EQ-5D-5L dimensions. It is known that younger members of the general population have an average utility score closer to 1 than older individuals; therefore, it is conceivable that at such a young age children may score this highly [42]. However, there are widely-accepted challenges associated with the assessment of HRQoL in children of a young age [43]. A high score may also reflect a potential ceiling effect of the EQ-5D measure for children with CLN2 disease, which warrants further study.

Patients with a CLN2 Clinical Rating Scale score of 6 are considered to have motor-language function similar to children of the same age without CLN2 disease. The utility values for this disease stage did not differ considerably between vignettes describing patients who had received cerliponase alfa and those who had not, likely reflecting these comparable motor-language functions. A slight reduction in utility values for vignettes describing patients treated with cerliponase alfa was observed (although not statistically significant). This could be due to the disutility associated with receiving treatment, which is administered by intracerebroventricular infusion every two weeks. Conversely, in the disease stages represented by a CLN2 Clinical Rating Scale score of 5 through to 0 + VL + PC, utility values for vignettes describing patients treated with cerliponase alfa were higher than for those treated with standard care. This suggests that within the same disease stage, treatment with cerliponase alfa may improve HRQoL compared with standard care, although these findings should be interpreted with caution.

The relative improvement in HRQoL, as indicated by the difference in utility values of treated and untreated patients, is consistent with observed clinical outcomes; in patients treated with cerliponase alfa for up to 141 weeks, it was observed that there was a significant attenuation in the rate of decline in CLN2 Clinical Rating Scale score over 48 weeks, and that this treatment effect persisted [16, 19]. Analyses of the individual dimensions of the proxy EQ-5D indicated that the difference in mean utility values of patients treated with cerliponase alfa and standard care may be driven primarily by differences in the pain/discomfort dimension, and to some extent the anxiety/depression dimension. For the mobility, self-care, and usual activities dimensions, mean levels were consistently lower (representing lower impairment) for patients treated with cerliponase alfa than for those treated with standard care, although it should be noted that statistical significance testing was not conducted during the dimension analysis.

The most noticeable differences in the anxiety/depression dimension occurred in the latter disease stages. A potential cause for this is a difference in the frequencies of GTCSs as described in the equivalent cerliponase alfa and standard care vignettes. For example, at CLN2 Clinical Rating Scale scores of 2 and 3 the vignettes describe the standard care patients as having six GTCSs per year, as compared to the cerliponase alfa-treated patients who only experience one GTCS per year. In these disease stages, differences of 1.0 and 0.7 in the mean anxiety/depression dimension score, respectively, were observed. This could suggest that the reduced GTCS frequency assumed for cerliponase alfa-treated patients has a measurable impact on HRQoL. Correspondingly, HRQoL has been observed to decrease with increasing GTCS frequency in adults with epilepsy [44]. However, it should be noted that a difference of 0.8 in the mean anxiety/depression dimension scores was seen for disease stage 0 + VL + PC, where the vignettes do not describe a difference in GTCS frequencies. This suggests that other aspects of CLN2 disease may also impact anxiety, and therefore HRQoL, which may lead to the observed differences between utility scores for the standard care and cerliponase alfa vignettes.

More generally, the assessment of anxiety (and similarly pain) may be problematic when conducted by an observer, as indicators linked with these EQ-5D dimensions (e.g. crying, restlessness) may be caused by brain involvement or dystonia rather than being reflective of anxiety itself. In addition, these indicators are likely to be similar for both pain/discomfort and anxiety/depression EQ-5D dimensions; changes observed in the pain experienced by CLN2 patients may be partly responsible for changes observed in anxiety, and vice versa, and therefore it may be difficult for an observer to establish causality behind indicators linked with these dimensions. The vignettes in this study were worded to ensure that clinicians could objectively assess a child’s anxiety and pain based on their experience with CLN2 patients. However, it should be noted that clinician perspectives on a child’s level of anxiety may also be altered by environmental factors such as location (children can become very upset when attending appointments in a hospital, for instance) and timing (e.g. soon after treatment infusion).

In the final disease stages, EQ-5D-5L and EQ-5D-3L utility values for vignettes describing patients treated with standard care fell below zero, with negative values indicating that death would be preferable to living in those disease states. All EQ-5D utilities were derived from published UK value sets (i.e. a set of weights for each of the levels of EQ-5D dimensions, which were determined by the UK general public) [45]. Based on previously published literature, it is reasonable to obtain negative utility values; negative utility values have been observed in the latter stages of other neurological diseases including dementia with Lewy bodies, stroke, multiple sclerosis and myasthenia gravis [46–49]. Whilst obtaining negative utility values may be reasonable, it cannot be assumed that a patient would directly rate their health in the latter disease states as being worse than death [50]. Direct elicitation of utilities may address this but, due to the nature of this paediatric neurodegenerative condition, was considered to be an inappropriate method for this case study.

A major limitation of the study was the absence of blinding to the intervention, which, although documented in previous studies [31, 32], has the potential to introduce bias when completing questionnaires based on vignette content. As described above, wording used in the vignettes differed between those describing patients receiving cerliponase alfa and those describing untreated patients, as there was a need to accurately convey the nature of administration of cerliponase alfa (surgical implantation of ventricular reservoir under the scalp) in the vignettes due to the impact it would likely have on patients’ HRQoL (Supplementary Information 1). In addition, results from the pivotal clinical trials for cerliponase alfa (Studies 190–201 and 190–202) and input from expert clinicians suggested that symptoms affecting domains of the CLN2 Disease Rating Scale in addition to Motor and Language differ between untreated patients and those treated with cerliponase alfa, and subsequently, the vignettes were aligned with these findings [19]. For example, symptoms such as myoclonus, spasticity and disease-related pain/distress are referred to as ‘minimal’ in vignettes describing patients receiving cerliponase alfa treatment, as opposed to their standard care counterparts, whilst the number of tonic–clonic seizures experienced also differs as described above. The subsequent difference in wording between the vignettes in order to reflect this meant that blinding to treatment was not possible. Clinicians may therefore have been more likely to suggest a higher HRQoL score based on their prior knowledge of the treatment effects. Despite this risk of bias it was determined that on balance, the alternative approach (i.e. the application of a uniform set of vignettes for treated and untreated patients) would not address the differential clinical outcomes observed in clinical practice, and would be methodologically flawed [51].

In the literature, there does not appear to be a consistent method for the development and validation of vignettes, however the input of multiple clinicians and/or patient groups is frequently used [31, 32, 52]. In line with this, validation of the vignettes in this study was conducted by an expert clinician and patient organisation. It is acknowledged that validation by a group of experts as opposed to a single clinician would have been preferable, however clinical experts with knowledge of CLN2 disease and treatment with standard care or cerliponase alfa were asked to complete the EQ-5D questionnaire. Given the rare nature of this condition, it was deemed more appropriate to use a single expert for vignette validation, to maximise the number of clinical experts available to complete the questionnaire without bias. The results observed were comparable when the responses of the clinical expert involved in validating the vignettes were removed from the analyses.

Given the complexity of the disease and treatment delivery, it was considered appropriate to exclusively use experts with suitable experience for the study, of which there are a small number, rather than a greater number of individuals with more limited experience. This may have led to a reduction in the generalisability of results presented here, although participants were selected from three European centres, with the aim that the geographical distribution would reduce any potential bias resulting from the small sample size. Given this smaller sample size, ICC values were calculated to test whether any single participant’s answers were considerably different from other participants’ responses. These demonstrated excellent inter-rater agreement [27], providing further evidence that the methodology of this study was robust.

In addition to those listed above, further challenges associated with the elicitation of disease stage utilities in conditions such as CLN2 disease may subject the study to limitation. For example, it is acknowledged that any limitations associated with the wording of the CLN2 Clinical Rating Scale, which was used in this study to define the various disease stages, will translate to this study. The CLN2 Clinical Rating Scale was applied in this study as it is well-accepted within the clinical community and allowed for the alignment of participating clinicians in their interpretation of the vignettes, particularly with respect to the predominant symptoms of CLN2 disease. However, the scale does not account for all aspects of the disease, such as other seizure types experienced in addition to GTCSs, and other quantifiable individual traits absent from the vignettes. It was expected that clinicians would have interpreted the vignettes based on their extensive experience of treating typical, ‘real-world’ patients with CLN2 disease and would have considered these broader aspects of the condition, thereby mitigating the impact of this limitation.

This study has successfully obtained utility values for nine different disease stages deemed to accurately capture disease progression in patients treated with cerliponase alfa and those treated with standard care. This expands upon the previously collected utility data for CLN2 disease, from Study 190–202 [17], by providing a more comprehensive overview of how HRQoL changes during the disease course, and also allows for direct comparison between patients treated with cerliponase alfa or standard care [17]. Data such as these can be used to support timely access to rare disease treatments, for which there is often limited availability of epidemiological and natural history data, as well as a paucity of information on the cost burden of disease available when orphan pharmaceutical products are launched [53].

## Conclusions

In summary, the results of this study highlight the severely detrimental effect CLN2 disease has on HRQoL, whilst acknowledging the potential benefits to HRQoL of early treatment with cerliponase alfa. Whilst subject to a number of limitations imposed by the nature of CLN2 disease and administration method of cerliponase alfa, this study has demonstrated an appropriate and effective methodology for eliciting utility values in an ultra-rare, paediatric neurodegenerative disease. It is recommended that future research builds on the valuable perspectives of both clinicians and families caring for children with CLN2 disease, thus allowing for further verification of these findings.

## Supplementary Information

**Additional file 1.** Supplementary Information.

## Data Availability

The datasets supporting the conclusions of this article are included within the article and its additional files.
