# Supplementary material for: Investigating health-related quality of life in rare diseases: a case study in utility value determination for patients with CLN2 disease (neuronal ceroid lipofuscinosis type 2)
Source: Orphanet J Rare Dis. 2021 May 12;16:217. doi: 10.1186/s13023-021-01829-x (PMC8117322; doi:10.1186/s13023-021-01829-x)
Supplement: Supplementary file 1 — Additional file 1. Supplementary Information. [file 13023_2021_1829_MOESM1_ESM.docx]

SUPPLEMENTARY INFORMATION

**Supplementary Information 1:** Vignettes

Standard Care Vignettes

CLN2 Clinical Rating Scale score 6, treated with standard care without cerliponase alfa (disease stage 6)

The patient is a child that has normal gait, no prominent ataxia, and doesn’t suffer from pathologic falls. These features correspond to a Motor score of 3 on the CLN2 Clinical Rating Scale. They have apparently normal language levels and are intelligible for their age. These features correspond to a Language score of 3 on the CLN2 Clinical Rating Scale. Their vision is normal. They have epilepsy, which is managed using anti-epileptic medications, but experience one generalised tonic-clonic seizure per year. They don’t experience disease-related pain/distress, dystonia, or myoclonus. They are not using a feeding tube, and they have normal social interactions.

CLN2 Clinical Rating Scale score 5, treated with standard care without cerliponase alfa (disease stage 5)

The patient is a child that has an independent gait, but obvious instability, and may have intermittent falls. These features correspond to a Motor score of 2 on the CLN2 Clinical Rating Scale. They have apparently normal language levels and are intelligible for their age. These features correspond to a Language score of 3 on the CLN2 Clinical Rating Scale. Their vision is normal. They have epilepsy, which is managed using anti-epileptic medications, but experience three generalised tonic-clonic seizures per year. They don’t experience disease-related pain/distress, dystonia, or myoclonus. They are using a feeding tube and they have relatively normal social interactions.

CLN2 Clinical Rating Scale score 4, treated with standard care without cerliponase alfa (disease stage 4)

The patient is a child that has an independent gait, but obvious instability, and may have intermittent falls. These features correspond to a Motor score of 2 on the CLN2 Clinical Rating Scale. Their language is limited for their age. This corresponds to a Language score of 2 on the CLN2 Clinical Rating Scale. Their vision is normal. They have epilepsy, which is managed using anti-epileptic medications, but experience six generalised tonic-clonic seizures per year. They don’t experience dystonia, but do experience myoclonus and spasticity, which cause disease-related pain/distress. They are using a feeding tube, and they have relatively normal social interactions.

CLN2 Clinical Rating Scale score 3, treated with standard care without cerliponase alfa (disease stage 3)

The patient is a child that requires external assistance to walk. This corresponds to a Motor score of 1 on the CLN2 Clinical Rating Scale. Their language is limited for their age. This corresponds to a Language score of 2 on the CLN2 Clinical Rating Scale. They experience problems recognising objects at distance. They have epilepsy, which is managed using anti-epileptic medications, but experience six generalised tonic-clonic seizures per year. They don’t experience dystonia, but do experience myoclonus and spasticity, which cause disease-related pain/distress. They are using a feeding tube, and they have some difficulty with social interactions.

CLN2 Clinical Rating Scale score 2, treated with standard care without cerliponase alfa (disease stage 2)

The patient is a child that requires external assistance to walk. This corresponds to a Motor score of 1 on the CLN2 Clinical Rating Scale. The patient is hardly understandable. This corresponds to a Language score of 1 on the CLN2 Clinical Rating Scale. They have problems recognising objects at distance. They have epilepsy, which is managed using anti-epileptic medications, but experience six generalised tonic-clonic seizures per year. They experience dystonia, myoclonus, and spasticity, which cause disease-related pain/distress. They are using a feeding tube, and they have moderate difficulty with social interactions.

CLN2 Clinical Rating Scale score 1, treated with standard care without cerliponase alfa (disease stage 1)

The patient is a child that requires external assistance to walk. This corresponds to a Motor score of 1 on the CLN2 Clinical Rating Scale. The patient is not understandable, with no intelligible words. This corresponds to a Language score of 0 on the CLN2 Clinical Rating Scale. They can only recognise objects right in front of them. They have epilepsy, which is managed using anti-epileptic medications, but experience six generalised tonic-clonic seizures per year. They experience dystonia, myoclonus, and spasticity, which cause disease-related pain/distress. They are using a feeding tube, and have severe difficulty with social interactions.

CLN2 Clinical Rating Scale score 0, treated with standard care without cerliponase alfa (disease stage 0)

The patient is a child that cannot walk or crawl. This corresponds to a Motor score of 0 on the CLN2 Clinical Rating Scale. The patient is not understandable, with no intelligible words. This corresponds to a Language score of 0 on the CLN2 Clinical Rating Scale. They are functionally blind. They have epilepsy, which is managed using anti-epileptic medications. They do not experience generalised tonic-clonic seizures. They experience, dystonia, myoclonus, and spasticity, which cause disease-related pain/distress. They are using a feeding tube, and have extreme difficulty with social interactions.

CLN2 Clinical Rating Scale score 0, with vision loss, treated with standard care without cerliponase alfa (disease stage 0+VL)

The patient is a child that has lost their ability to walk or crawl. This corresponds to a Motor score of 0 on the CLN2 Clinical Rating Scale. They have no intelligible words or vocalisations. This corresponds to a Language score of 0 on the CLN2 Clinical Rating Scale. They have complete vision loss. They have epilepsy, which is managed using anti-epileptic medications. They do not experience generalised tonic-clonic seizures. They experience dystonia, myoclonus, and spasticity, which cause disease-related pain/distress. They are using a feeding tube, and are unable to interact socially.

CLN2 Clinical Rating Scale score 0, requiring palliative care, treated with standard care without cerliponase alfa (disease stage 0+VL+PC)

The patient is a child that has lost their ability to walk or crawl. This corresponds to a Motor score of 0 on the CLN2 Clinical Rating Scale. They have no intelligible words or vocalisations. This corresponds to a Language score of 0 on the CLN2 Clinical Rating Scale. They have complete vision loss. They have epilepsy, which is managed using anti-epileptic medications. They do not experience generalised tonic-clonic seizures. They experience dystonia, myoclonus, and spasticity, which cause disease-related pain/distress. They are using a feeding tube, require secretion management, and have significant respiratory assistance requirements, requiring a ventilator day and night. They are incontinent of bowel and bladder, and are unable to interact socially.

Cerliponase alfa Vignettes

CLN2 Clinical Rating Scale score 6, treated with cerliponase alfa (disease stage 6)

The patient is a child that has normal gait, no prominent ataxia, and doesn’t suffer from pathologic falls. These features correspond to a Motor score of 3 on the CLN2 Clinical Rating Scale. They have apparently normal language levels and are intelligible for their age. These features correspond to a Language score of 3 on the CLN2 Clinical Rating Scale. Their vision is normal. They have epilepsy, which is managed using anti-epileptic medications, but experience one generalised tonic-clonic seizure per year. They don’t experience disease-related pain/distress, dystonia, or myoclonus. They are not using a feeding tube, and they have normal social interactions. They are currently being treated with cerliponase alfa, which is administered every other week by intracerebroventricular infusion for four hours.

CLN2 Clinical Rating Scale score 5, treated with cerliponase alfa (disease stage 5)

The patient is a child that has an independent gait, but obvious instability, and may have intermittent falls. These features correspond to a Motor score of 2 on the CLN2 Clinical Rating Scale. They have apparently normal language levels and are intelligible for their age. These features correspond to a Language score of 3 on the CLN2 Clinical Rating Scale. Their vision is normal. They have epilepsy, which is managed using anti-epileptic medications, but experience one generalised tonic-clonic seizure per year. They don’t experience disease-related pain/distress, dystonia, or myoclonus. They are not using a feeding tube, and they have relatively normal social interactions. They are currently being treated with cerliponase alfa, which is administered every other week by intracerebroventricular infusion for four hours.

CLN2 Clinical Rating Scale score 4, treated with cerliponase alfa (disease stage 4)

The patient is a child that has an independent gait, but obvious instability, and may have intermittent falls. These features correspond to a Motor score of 2 on the CLN2 Clinical Rating Scale. Their language is limited for their age. This corresponds to a Language score of 2 on the CLN2 Clinical Rating Scale. Their vision is normal. They have epilepsy, which is managed using anti-epileptic medications, but experience one generalised tonic-clonic seizure per year. They don’t experience dystonia, but do experience minimal myoclonus and minimal spasticity, which cause minimal disease-related pain/distress. They are not using a feeding tube, and they have relatively normal social interactions. They are currently being treated with cerliponase alfa, which is administered every other week by intracerebroventricular infusion for four hours.

CLN2 Clinical Rating Scale score 3, treated with cerliponase alfa (disease stage 3)

The patient is a child that requires external assistance to walk. This corresponds to a Motor score of 1 on the CLN2 Clinical Rating Scale. Their language is limited for their age. This corresponds to a Language score of 2 on the CLN2 Clinical Rating Scale. They experience problems recognising objects at distance. They have epilepsy, which is managed using anti-epileptic medications, but experience one generalised tonic-clonic seizure per year. They don’t experience dystonia, but do experience minimal myoclonus and minimal spasticity, which cause minimal disease-related pain/distress. They are using a feeding tube, and they have some difficulty with social interactions. They are currently being treated with cerliponase alfa, which is administered every other week by intracerebroventricular infusion for four hours.

CLN2 Clinical Rating Scale score 2, treated with cerliponase alfa (disease stage 2)

The patient is a child that requires external assistance to walk. This corresponds to a Motor score of 1 on the CLN2 Clinical Rating Scale. The patient is hardly understandable. This corresponds to a Language score of 1 on the CLN2 Clinical Rating Scale. They have problems recognising objects at distance. They have epilepsy, which is managed using anti-epileptic medications, but experience one generalised tonic-clonic seizure per year. They experience dystonia, minimal myoclonus and minimal spasticity, which cause minimal disease-related pain/distress. They are using a feeding tube, and they have some difficulty with social interactions. They are currently being treated with cerliponase alfa, which is administered every other week by intracerebroventricular infusion for four hours.

CLN2 Clinical Rating Scale score 1, treated with cerliponase alfa (disease stage 1)

The patient is a child that requires external assistance to walk. This corresponds to a Motor score of 1 on the CLN2 Clinical Rating Scale. The patient is not understandable, with no intelligible words. This corresponds to a Language score of 0 on the CLN2 Clinical Rating Scale. They can only recognise objects right in front of them. They have epilepsy, which is managed using anti-epileptic medications, but experience one generalised tonic-clonic seizure per year. They experience, dystonia, minimal myoclonus, and minimal spasticity, which cause minimal disease-related pain/distress. They are using a feeding tube, and have moderate difficulty with social interactions. They are currently being treated with cerliponase alfa, which is administered every other week by intracerebroventricular infusion for four hours.

CLN2 Clinical Rating Scale score 0, treated with cerliponase alfa (disease stage 0)

The patient is a child that cannot walk or crawl. This corresponds to a Motor score of 0 on the CLN2 Clinical Rating Scale. The patient is not understandable, with no intelligible words. This corresponds to a Language score of 0 on the CLN2 Clinical Rating Scale. They are functionally blind. They have epilepsy, which is managed using anti-epileptic medications. They do not experience generalised tonic-clonic seizures. They experience dystonia, minimal myoclonus, and minimal spasticity, which cause minimal disease-related pain/distress. They are using a feeding tube, and have serious difficulty with social interactions. They are currently being treated with cerliponase alfa, which is administered every other week by intracerebroventricular infusion for four hours.

CLN2 Clinical Rating Scale score 0, with vision loss, treated with cerliponase alfa (disease stage 0+VL)

The patient is a child that has lost their ability to walk or crawl. This corresponds to a Motor score of 0 on the CLN2 Clinical Rating Scale. They have no intelligible words or vocalisations. This corresponds to a Language score of 0 on the CLN2 Clinical Rating Scale. They have complete vision loss. They have epilepsy, which is managed using anti-epileptic medications. They do not experience generalised tonic-clonic seizures. They experience dystonia, minimal myoclonus, and minimal spasticity which cause minimal disease-related pain/distress. They are using a feeding tube, require secretion management, and are unable to interact socially. They are currently being treated with cerliponase alfa, which is administered every other week by intracerebroventricular infusion for four hours.

CLN2 Clinical Rating Scale score 0, requiring palliative care, treated with cerliponase alfa (disease stage 0+VL+PC)

The patient is a child that has lost their ability to walk or crawl. This corresponds to a Motor score of 0 on the CLN2 Clinical Rating Scale. They have no intelligible words or vocalisations. This corresponds to a Language score of 0 on the CLN2 Clinical Rating Scale. They have complete vision loss. They have epilepsy, which is managed using anti-epileptic medications. They do not experience generalised tonic-clonic seizures. They experience dystonia, minimal myoclonus, and minimal spasticity, which cause disease-related pain/distress. They are using a feeding tube, require secretion management, have significant respiratory assistance requirements, requiring a ventilator day and night, and are unable to interact socially. They are incontinent of bowel and bladder, and are currently being treated with cerliponase alfa, which is administered every other week by intracerebroventricular infusion for four hours.

**Supplementary Information 2:** Supplementary tables and figures


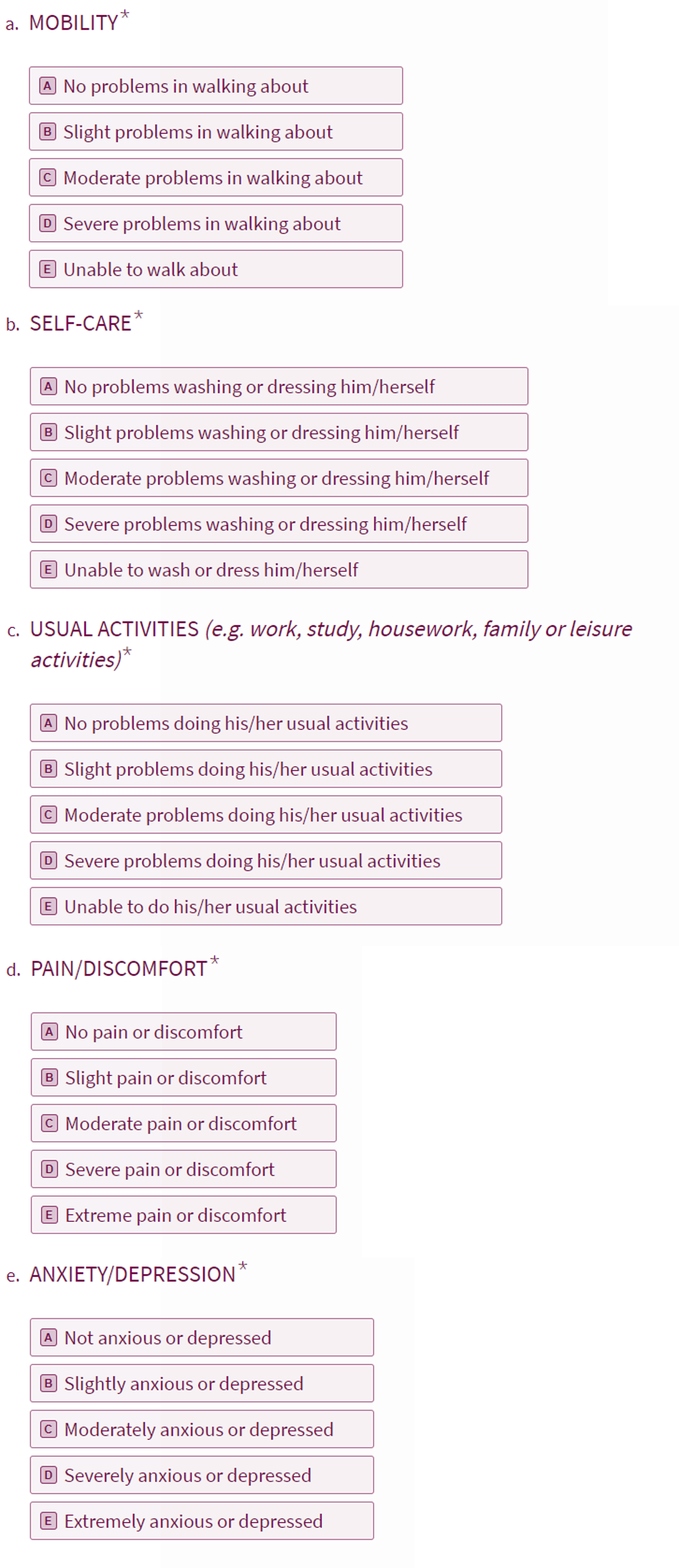
Additional file 1: Figure S1. EQ-5D-5L proxy version 2 questionnaire

The questionnaire was generated in Typeform and validated by EuroQol. For comparison, questions in the mobility section of the non-proxy EQ-5D-5L read as follows: a) I have no problems in walking about; b) I have slight problems in walking about; c) I have moderate problems in walking about; d) I have severe problems in walking about; e) I am unable to walk about.^1^

**Additional file 1: Table S1.** Scoring for Motor and Language Function on the CLN2 Clinical Rating Scale.

| **Score** | **Functional Description** | |
| --- | --- | --- |
|  | **Motor Domain** | **Language Domain** |
| 3 | Has grossly normal gait; no prominent ataxia, no pathologic falls | Has apparently normal language that is intelligible and grossly age-appropriate, with no decline noted |
| 2 | Has independent gait as defined by ability to walk without support for 10 steps; obvious instability and possibly intermittent falls | Has language that has recognisable abnormalities but includes some intelligible words; may form short sentences to convey concepts, requests or needs |
| 1 | Requires external assistance to walk or can only crawl | Has language that is hard to understand with few intelligible words |
| 0 | Can no longer walk or crawl | Has no intelligible words or vocalisations |

Adapted from Schulz *et al*. 2018.

Additional file 1: Table S2. Utility values for cerliponase alfa-treated patients, excluding responses from clinical expert involved in vignette validation

|  | **Cerliponase alfa** | | | | |
| --- | --- | --- | --- | --- | --- |
| Disease stage | **Mean value** | Standard error | Median value | Minimum value | Maximum value |
| 6 | **0.989** | 0.011 | 1.000 | 0.924 | 1.000 |
| 5 | **0.851** | 0.009 | 0.846 | 0.825 | 0.901 |
| 4 | **0.750** | 0.021 | 0.767 | 0.642 | 0.801 |
| 3 | **0.478** | 0.065 | 0.451 | 0.302 | 0.666 |
| 2 | **0.447** | 0.062 | 0.420 | 0.267 | 0.666 |
| 1 | **0.341** | 0.061 | 0.317 | 0.167 | 0.652 |
| 0 | **0.116** | 0.064 | 0.167 | -0.213 | 0.282 |
| 0 + VL | **0.106** | 0.073 | 0.181 | -0.281 | 0.282 |
| 0 + VL + PC | **0.093** | 0.074 | 0.167 | -0.281 | 0.268 |

Disease progression increases with decreasing disease stage. Utility values are given on a scale where 1 is equivalent to perfect health, and 0 equivalent to death. PC: palliative care; VL: vision loss.

Additional file 1: Table S3. Utility values for standard care patients, excluding responses from clinical expert involved in vignette validation

|  | **Standard Care** | | | | |
| --- | --- | --- | --- | --- | --- |
| Disease stage | **Mean value** | Standard error | Median value | Minimum value | Maximum value |
| 6 | **1.000** | 0.000 | 1.000 | 1.000 | 1.000 |
| 5 | **0.810** | 0.037 | 0.846 | 0.608 | 0.901 |
| 4 | **0.651** | 0.041 | 0.652 | 0.447 | 0.801 |
| 3 | **0.313** | 0.078 | 0.359 | -0.102 | 0.522 |
| 2 | **0.159** | 0.074 | 0.243 | -0.137 | 0.329 |
| 1 | **0.174** | 0.059 | 0.228 | -0.137 | 0.329 |
| 0 | **-0.130** | 0.055 | -0.198 | -0.276 | 0.073 |
| 0 + VL | **-0.063** | 0.067 | -0.027 | -0.276 | 0.206 |
| 0 + VL + PC | **-0.101** | 0.072 | -0.213 | -0.281 | 0.191 |

Disease progression increases with decreasing disease stage. Utility values are given on a scale where 1 is equivalent to perfect health, and 0 equivalent to death. PC: palliative care; VL: vision loss.

**Additional file 1: Figure S2.** EQ-5D-5L utility values excluding responses from clinical expert involved in vignette validation


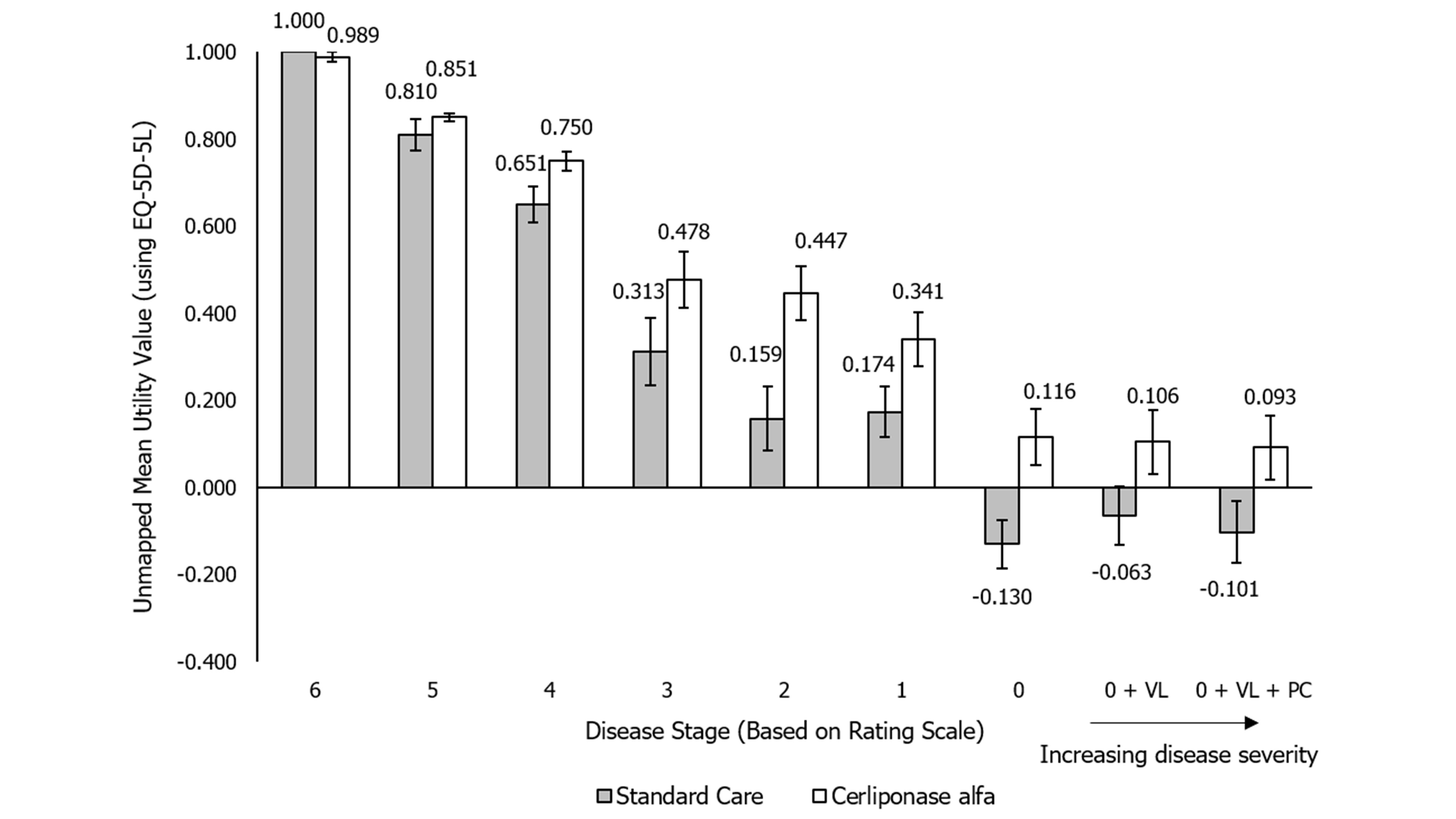


Mean values ± 1 standard error are shown on the chart. Utility values are given on a scale where 1 is equivalent to perfect health, and 0 equivalent to death. VL, vision loss; PC, palliative care.

Additional file 1: Table S4. Utility values for cerliponase alfa-treated patients, excluding responses from expert involved in vignette validation, mapped to EQ-5D-3L.

|  | **Cerliponase alfa** | | | | |
| --- | --- | --- | --- | --- | --- |
| Disease stage | **Mean value** | Standard error | Median value | Minimum value | Maximum value |
| 6 | **0.983** | 0.017 | 1.000 | 0.879 | 1.000 |
| 5 | **0.764** | 0.013 | 0.747 | 0.747 | 0.836 |
| 4 | **0.635** | 0.021 | 0.648 | 0.541 | 0.710 |
| 3 | **0.451** | 0.039 | 0.504 | 0.325 | 0.555 |
| 2 | **0.405** | 0.061 | 0.431 | 0.101 | 0.555 |
| 1 | **0.199** | 0.087 | 0.196 | -0.166 | 0.531 |
| 0 | **-0.174** | 0.035 | -0.166 | -0.352 | -0.071 |
| 0 + VL | **-0.208** | 0.068 | -0.151 | -0.594 | -0.071 |
| 0 + VL + PC | **-0.221** | 0.066 | -0.166 | -0.594 | -0.095 |

Disease progression increases with decreasing disease stage. Utility values are given on a scale where 1 is equivalent to perfect health, and 0 equivalent to death. Summary values were produced from the returned EQ-5D-5L questionnaires and results were mapped to equivalent EQ-5D-3L utility values using the Van Hout algorithm. PC: palliative care; VL: vision loss.

Additional file 1: Table S5. Utility values for standard care patients, excluding responses from expert involved in vignette validation, mapped to EQ-5D-3L.

|  | **Standard Care** | | | | |
| --- | --- | --- | --- | --- | --- |
| Disease stage | **Mean value** | Standard error | Median value | Minimum value | Maximum value |
| 6 | **1.000** | 0.000 | 1.000 | 1.000 | 1.000 |
| 5 | **0.729** | 0.035 | 0.747 | 0.544 | 0.836 |
| 4 | **0.539** | 0.041 | 0.531 | 0.353 | 0.710 |
| 3 | **0.328** | 0.065 | 0.310 | 0.036 | 0.518 |
| 2 | **0.106** | 0.062 | 0.100 | -0.087 | 0.310 |
| 1 | **0.075** | 0.030 | 0.100 | -0.086 | 0.155 |
| 0 | **-0.359** | 0.044 | -0.352 | -0.510 | -0.200 |
| 0 + VL | **-0.310** | 0.047 | -0.352 | -0.510 | -0.127 |
| 0 + VL + PC | **-0.360** | 0.059 | -0.352 | -0.594 | -0.151 |

Disease progression increases with decreasing disease stage. Utility values are given on a scale where 1 is equivalent to perfect health, and 0 equivalent to death. Summary values were produced from the returned EQ-5D-5L questionnaires and results were mapped to equivalent EQ-5D-3L utility values using the Van Hout algorithm. PC: palliative care; VL: vision loss.

Additional file 1: Figure S3. Mean utility values with response from clinical expert involved in vignette validation excluded and results mapped to the EQ-5D-3L.


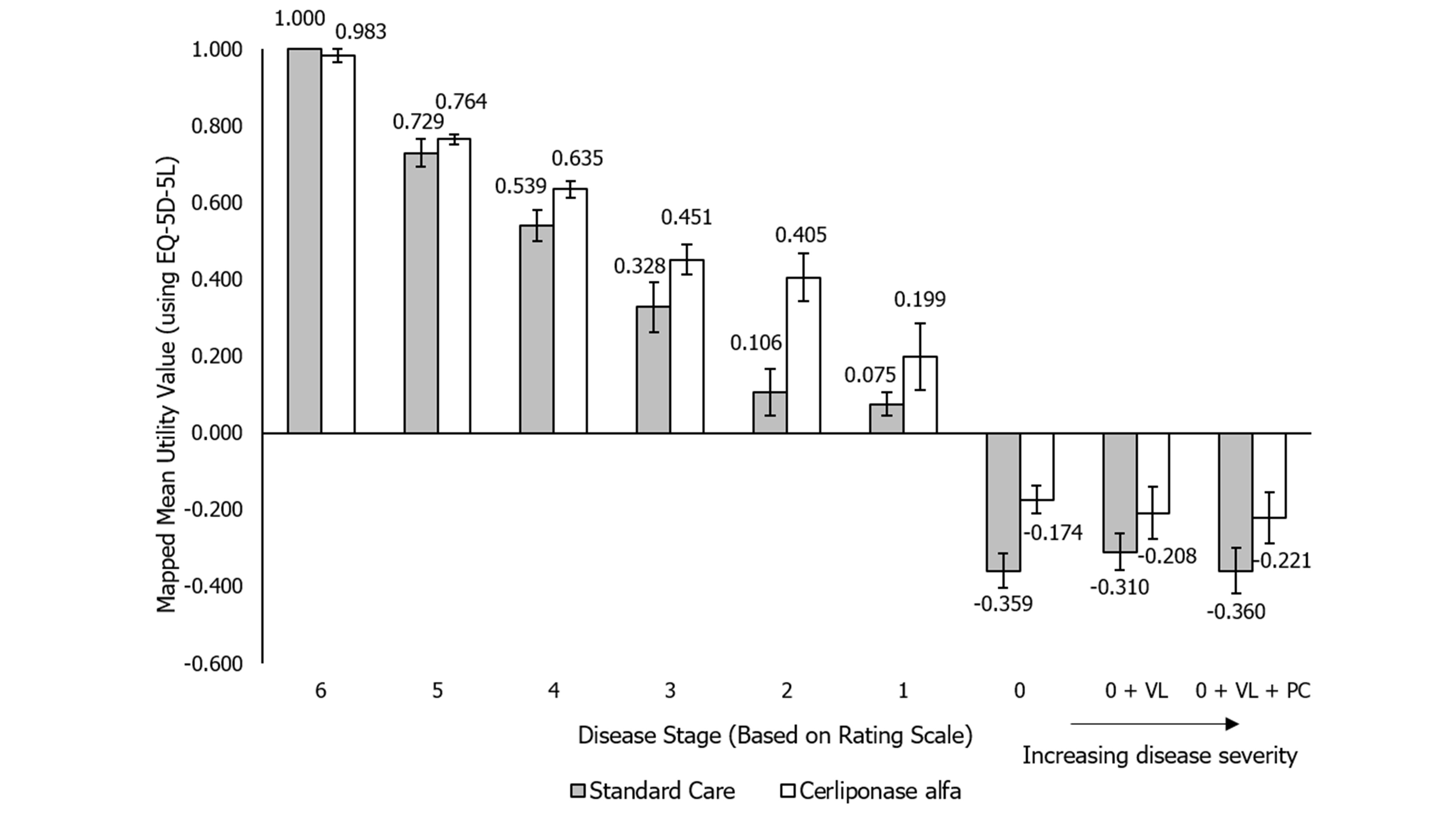


Results were mapped to EQ-5D-3L utility values using the Van Hout algorithm. Mean values ± 1 standard error are shown on the chart. Utility values are given on a scale where 1 is equivalent to perfect health, and 0 equivalent to death. VL, vision loss; PC, palliative care.

**Additional file 1: Table S6.** Utility values of cerliponase alfa-treated patients using the EQ-5D-5L German value set.

|  | **Cerliponase alfa** | | | | |
| --- | --- | --- | --- | --- | --- |
| Disease stage | **Mean value** | Standard error | Median value | Minimum value | Maximum value |
| 6 | 0.996 | 0.004 | 1.000 | 0.970 | 1.000 |
| 5 | 0.893 | 0.004 | 0.888 | 0.888 | 0.924 |
| 4 | 0.810 | 0.015 | 0.822 | 0.714 | 0.853 |
| 3 | 0.612 | 0.051 | 0.613 | 0.424 | 0.766 |
| 2 | 0.572 | 0.065 | 0.576 | 0.305 | 0.766 |
| 1 | 0.391 | 0.068 | 0.448 | 0.116 | 0.714 |
| 0 | 0.117 | 0.075 | 0.142 | -0.341 | 0.311 |
| 0 + VL | 0.066 | 0.110 | 0.168 | -0.661 | 0.311 |
| 0 + VL + PC | -0.006 | 0.107 | 0.142 | -0.661 | 0.207 |

Disease progression increases with decreasing disease stage. Utility values are given on a scale where 1 is equivalent to perfect health, and 0 equivalent to death. PC: palliative care; VL: vision loss.

**Additional file 1: Table S7.** Utility values of standard care patients using the EQ-5D-5L German value set.

|  | **Standard care** | | | | |
| --- | --- | --- | --- | --- | --- |
| Disease stage | **Mean value** | Standard error | Median value | Minimum value | Maximum value |
| 6 | 1.000 | 0.000 | 1.000 | 1.000 | 1.000 |
| 5 | 0.863 | 0.028 | 0.888 | 0.674 | 0.924 |
| 4 | 0.730 | 0.038 | 0.748 | 0.521 | 0.853 |
| 3 | 0.430 | 0.083 | 0.480 | -0.085 | 0.669 |
| 2 | 0.191 | 0.084 | 0.288 | -0.197 | 0.372 |
| 1 | 0.147 | 0.058 | 0.201 | -0.165 | 0.292 |
| 0 | -0.326 | 0.057 | -0.323 | -0.549 | -0.097 |
| 0 + VL | -0.256 | 0.084 | -0.323 | -0.549 | 0.220 |
| 0 + VL + PC | -0.348 | 0.101 | -0.341 | -0.661 | 0.168 |

Disease progression increases with decreasing disease stage. Utility values are given on a scale where 1 is equivalent to perfect health, and 0 equivalent to death. PC: palliative care; VL: vision loss.


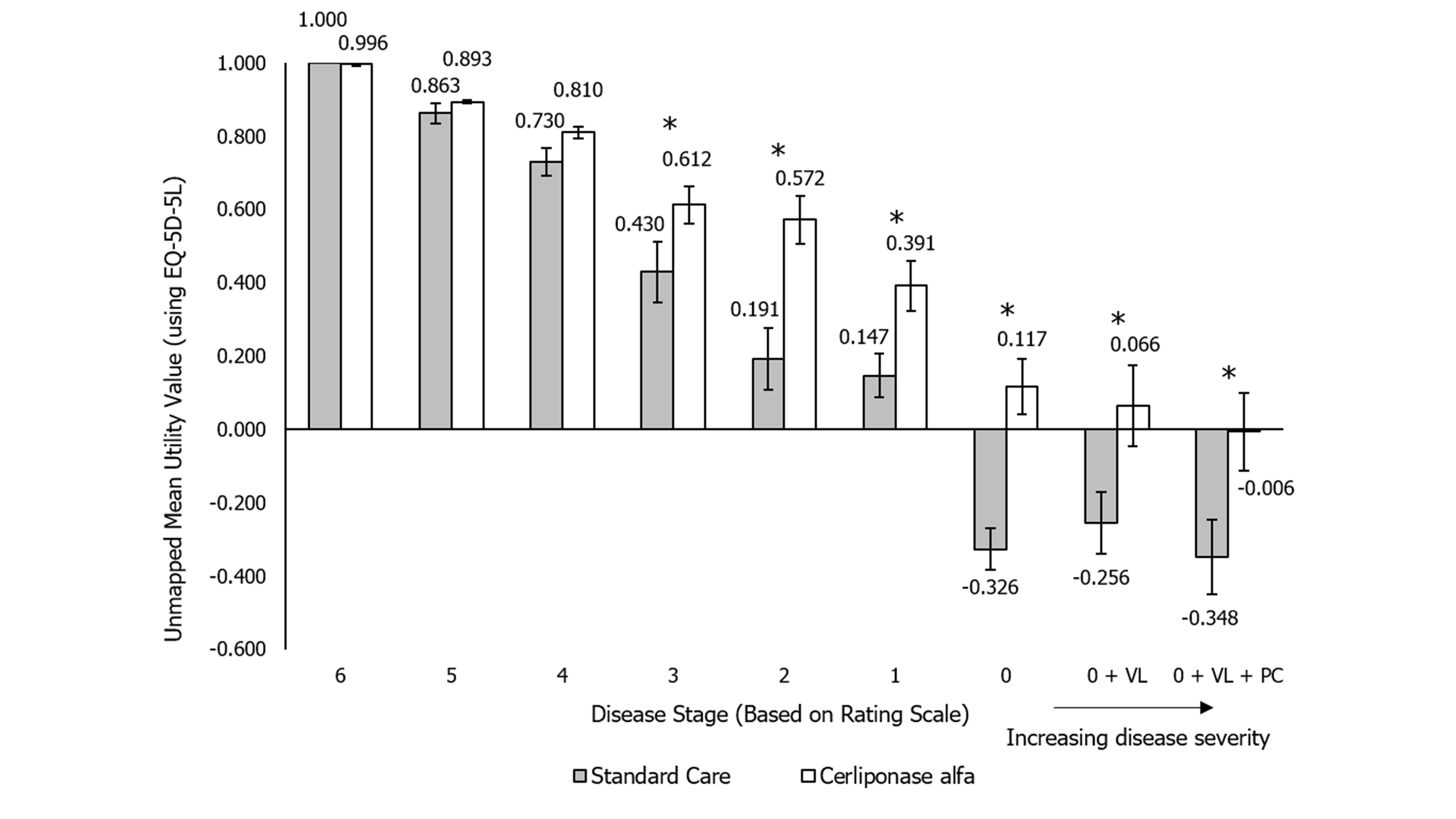
Additional file 1: Figure S4. Mean utility values across disease stages using the EQ-5D-5L German value set.

Mean values ± 1 standard error are shown on the chart. Utility values are given on a scale where 1 is equivalent to perfect health, and 0 equivalent to death. Asterisks (*) indicate a statistically significant difference between treatment with standard care and cerliponase alfa using a paired t-test, p<0.05. VL, vision loss; PC, palliative care.

**Additional file 1: Table S8.** Utility values of cerliponase alfa-treated patients using the EQ-5D-5L Spanish value set.

|  | **Cerliponase alfa** | | | | |
| --- | --- | --- | --- | --- | --- |
| Disease stage | **Mean value** | Standard error | Median value | Minimum value | Maximum value |
| 6 | 0.990 | 0.010 | 1.000 | 0.919 | 1.000 |
| 5 | 0.823 | 0.008 | 0.822 | 0.785 | 0.866 |
| 4 | 0.706 | 0.023 | 0.722 | 0.593 | 0.799 |
| 3 | 0.474 | 0.060 | 0.510 | 0.269 | 0.640 |
| 2 | 0.443 | 0.063 | 0.427 | 0.222 | 0.640 |
| 1 | 0.298 | 0.057 | 0.292 | 0.085 | 0.617 |
| 0 | 0.084 | 0.050 | 0.108 | -0.203 | 0.235 |
| 0 + VL | 0.053 | 0.074 | 0.120 | -0.416 | 0.235 |
| 0 + VL + PC | 0.041 | 0.072 | 0.096 | -0.416 | 0.212 |

Disease progression increases with decreasing disease stage. Utility values are given on a scale where 1 is equivalent to perfect health, and 0 equivalent to death. PC: palliative care; VL: vision loss.

**Additional file 1: Table S9.** Utility values of standard care patients using the EQ-5D-5L Spanish value set.

|  | **Standard care** | | | | |
| --- | --- | --- | --- | --- | --- |
| Disease stage | **Mean value** | Standard error | Median value | Minimum value | Maximum value |
| 6 | 1.000 | 0.000 | 1.000 | 1.000 | 1.000 |
| 5 | 0.782 | 0.032 | 0.822 | 0.576 | 0.866 |
| 4 | 0.631 | 0.039 | 0.636 | 0.420 | 0.799 |
| 3 | 0.302 | 0.062 | 0.363 | -0.065 | 0.489 |
| 2 | 0.140 | 0.057 | 0.213 | -0.142 | 0.300 |
| 1 | 0.139 | 0.041 | 0.172 | -0.083 | 0.300 |
| 0 | -0.164 | 0.050 | -0.187 | -0.339 | 0.068 |
| 0 + VL | -0.116 | 0.060 | -0.136 | -0.339 | 0.155 |
| 0 + VL + PC | -0.181 | 0.074 | -0.203 | -0.416 | 0.131 |

Disease progression increases with decreasing disease stage. Utility values are given on a scale where 1 is equivalent to perfect health, and 0 equivalent to death. PC: palliative care; VL: vision loss.

Additional file 1: Figure S5. Mean utility values across disease stages using the EQ-5D-5L Spanish value set.


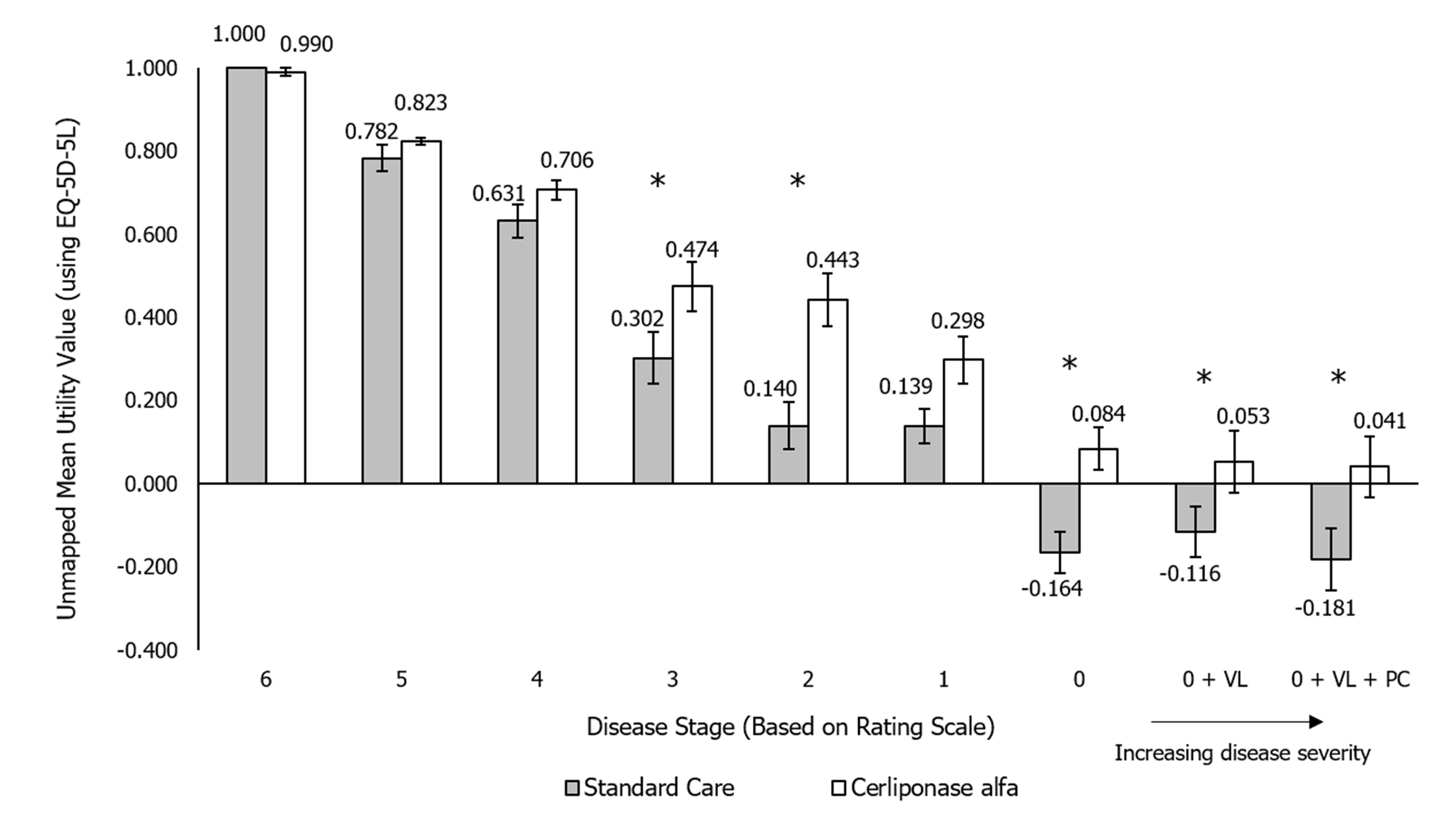


Mean values ± 1 standard error are shown on the chart. Utility values are given on a scale where 1 is equivalent to perfect health, and 0 equivalent to death. Asterisks (*) indicate a statistically significant difference between treatment with standard care and cerliponase alfa using a paired t-test, p<0.05. VL, vision loss; PC, palliative care.

**SUPPLEMENTARY REFERENCES**

1. EuroQoL. EQ-5D. Available at: <https://euroqol.org/wp-content/uploads/2016/10/Sample_UK__English__EQ-5D-5L_Paper_Self_complete_v1.0__ID_24700.pdf>. Last accessed 13.06.19
